# Clinicians’ Contributions to the Development of Coronary Artery Stents: A Qualitative Study of Transformative Device Innovation

**DOI:** 10.1371/journal.pone.0088664

**Published:** 2014-02-12

**Authors:** Aaron S. Kesselheim, Shuai Xu, Jerry Avorn

**Affiliations:** Program On Regulation, Therapeutics, And Law (PORTAL), Division of Pharmacoepidemiology and Pharmacoeconomics, Department of Medicine, Brigham and Women’s Hospital and Harvard Medical School, Boston, Massachusetts, United States of America; Istituto Clinico S. Ambrogio, Italy

## Abstract

**Background:**

Medical device innovation remains poorly understood, and policymakers disagree over how to incentivize early development. We sought to elucidate the components of transformative health care innovation by conducting an in-depth case study of development of a key medical device: coronary artery stents.

**Methods and Findings:**

We conducted semi-structured interviews with the innovators whose work contributed to the development of coronary artery stents who we identified based on a review of the regulatory, patent, and medical literature. Semi-structured interviews with each participant covered the interviewee’s personal involvement in coronary artery stent development, the roles of institutions and other individuals in the development process, the interplay of funding and intellectual property in the interviewee’s contribution, and finally reflections on lessons arising from the experience. Transcripts were analyzed using standard coding techniques and the constant comparative method of qualitative data analysis.

**Conclusions:**

We found that the first coronary artery stents emerged from three teams: Julio Palmaz and Richard Schatz, Cesare Gianturco and Gary Roubin, and Ulrich Sigwart. First, these individual physician-inventors saw the need for coronary artery stents in their clinical practice. In response, they developed prototypes with the support of academic medical centers leading to early validation studies. Larger companies entered afterwards with engineering support. Patents became paramount once the technology diffused. The case of coronary stents suggests that innovation policy should focus on supporting early physician-inventors at academic centers.

## Introduction

Medical device innovation is essential for the development of new diagnostics and treatments for a wide range of conditions. However, the emergence of transformative new medical devices has slowed in recent years [1], [2]. Strategies intended to stimulate development have been offered [3], [4], but a lack of a consensus about the sources of innovation has stymied effective policymaking. Indeed, despite the importance of breakthrough medical technologies, the process of developing them remains poorly understood [5]. For example, some legislators and industry advocates argue that the best way to promote important new device research and development is to reduce regulatory hurdles in the US [6]. For this reason, the 2012 FDA Safety and Innovation Act included a number of provisions intended to streamline review of new devices [7].

A few studies have investigated the origins of transformative medical devices [8]. Some have used patent records to document the prominent role of individual physician-inventors [9], [10]. However, the patent record may be incomplete because underlying discoveries may not have been patentable [11].

In the present analysis, we conducted a qualitative study of transformative medical device innovation through structured interviews with key innovators involved in the early development of coronary artery stents, a technology that revolutionized cardiology [5], [12], [13]. These devices are one of the most important medical device innovations in the last 30 years [14], and exhibited impressive diffusion from use in 5.4% of all percutaneous coronary procedures in 1994 to 69% in 1997 despite the lack of a specific Diagnosis Related Group code for stent implantation [15]. Our goal was to identify the roles individuals and institutions played in the inception and development of coronary artery stents, as well as the challenges faced by innovators and the determinants that led to technology’s successful implementation.

## Methods

A qualitative research approach makes it possible to investigate motivations, reflections, and outcomes in a small cohort of subjects who share a common experience [16]. We conducted semi-structured interviews with the innovators whose work contributed to the development of coronary artery stents; this technique has been used previously to address fundamental questions relating to the development and adoption of new medical technologies [8]. Based on a review of the regulatory, patent, and medical literature, we targeted 37 potentially relevant innovators whose contact information we could identify through public sources, including 6 high priority targets. Fourteen agreed—including all of our high priority targets—while 2 declined. We identified two additional participants through referrals. The study methodology was approved by the institutional review board at Brigham and Women’s Hospital. Participants provided verbal consent (written consent was not deemed necessary by the institutional review board), which was documented in the interviewer’s notes at the beginning of the interview.

Semi-structured interviews with each participant covered four main topic areas: the interviewee’s personal involvement in coronary artery stent development, the roles of institutions and other individuals in the development process, the interplay of funding and intellectual property in the interviewee’s contribution, and finally reflections on lessons arising from the experience. Participants were asked to proceed chronologically through idea conception, product development, testing, and approval. Next, participants were asked to assess how academic medical centers, various companies they interacted with, and government regulatory authorities were involved with the work at different stages in the development. Third, participants were asked to recall how each phase of the development process was funded (private vs. industry vs. government), and whether patents were sought to protect their intellectual contributions. Finally, participants were asked to consider the roles of individual initiative, environmental factors, serendipity vs. strategic planning, advances in science and technology, and clinical need.

Median time for telephone interviews with participants was 40 minutes (range: 23–75). Both investigators took notes during the interview, and interviews were recorded and later transcribed. Interview transcripts were analyzed using standard coding techniques [17] and the constant comparative method of qualitative data analysis [18]. Based on a subset of 3 randomly selected interviews, the investigators conducted independent analysis and developed separate coding schemes for organizing the data [19]. The coding schemes were then compared, discussed and reconciled (NVIVO software package, QSR International, Melbourne, Australia) to produce a final coding structure which consisting of 7 broad themes (with 92 specific codes): (1) antecedents of stent development, (2) timeline of stent development, (3) key contributors to stent development, (4) the role of intellectual property, (5) the role of academic medical centers, (6) the role of device companies, and (7) other key characteristics of the inventive process.

## Results

### Precedents for coronary artery stent innovation

Interviewees pointed to three antecedent developments that set the stage for the development of coronary artery stents. The first was the practice of dilating arteries using percutaneous angioplasty, pioneered by Charles Dotter, a radiologist at the Oregon Health and Science University in the 1960s. Dotter developed a graduated catheter system with dilation by means of progressively larger catheters. Dotter developed his early catheter prototypes with the aid of Cook Inc, a small company founded by an early medical entrepreneur, Bill Cook. The second major antecedent was the development of percutaneous transluminal coronary angioplasty (PTCA). Andreas Gruentzig, a German cardiologist who came to Emory University in 1980, helped pioneer angioplasty in the coronary arteries, primarily through the use of an inelastic balloon [20]. A final key development mentioned by participants was improvement in the manufacturing of catheters required to deliver stents to the coronary arteries. Several innovators cited John Simpson, a cardiologist at Stanford University, who introduced a new catheter system that vastly improved steerability. He founded ACS, a privately held medical device company, to commercialize catheters and guidewires.

### Coronary artery stent development

The first coronary artery stents emerged from three teams. Two were US-based, with one led by Julio Palmaz and Richard Schatz, and another by Cesare Gianturco and Gary Roubin. The third was European-based led by Ulrich Sigwart. Each of the three stents was developed with a different concept in mind and their structures were completely different.

**The Palmaz-Schatz stent.** Argentina-trained radiologist Julio Palmaz, attended a talk by Gruentzig at the Society of Interventional Radiology Meeting in New Orleans in 1978. On the taxicab ride back to the airport, Palmaz conceived of his initial concept of the stent. Palmaz soon began fashioning his slotted tube stents in his garage.

Palmaz moved to the University of Texas-San Antonio in 1980 to continue his work. With dedicated research time and laboratory space, Palmaz finished animal studies of his stent, which he presented at the Radiological Society of North America annual meetings in 1984 and 1985. In 1985, Palmaz met Richard Schatz, an interventional cardiologist conducting research at the Southwest Research Institute in San Antonio. Schatz made a modification to Palmaz’s design to improve the stent’s flexibility and introduced Palmaz to his friend Philip Romano, a restaurateur. Romano provided $250,000 in seed money and the three formed Expandable Grafts Partnership (EGP) in late 1985 and then filed the first patent application on the technology.

Prior to EGP, Palmaz unsuccessfully sought company partners. However, with more mature technology and a business partner, EGP licensed its intellectual property to Johnson & Johnson in 1986 for $10 million and a royalty percentage (6–9% on use in the coronaries and 3–6% for peripheral use based on gross sales). Johnson & Johnson provided engineering support for Palmaz and Schatz and organized and funded the pivotal trials for US premarket approval. Human experiments with the Palmaz-Schatz stent occurred in peripheral arteries in 1987 and coronary arteries in 1988. First sales of the stent came in Europe by 1988. The FDA initially rejected the first Palmaz-Schatz stent application in 1993. However, the team quickly reapplied and gained FDA approval in August 1994 for the elective use of the Palmaz-Schatz stent for restenosis on the basis of two pivotal trials (BENESTENT and STRESS).

**The Gianturco-Roubin stent.** Cesar Gianturco was an accomplished innovator in interventional radiology who did much of his work at the Carle Clinic in Urbana, Illinois before becoming a professor of experimental diagnostic surgery at the University of Texas MD Anderson Hospital [21]. Gianturco had a long history working with Cook Inc. having developed balloon-deployable metallic stents and intravascular filters for peripheral vessels [22]. With funding and engineering support from Cook, Gruentzig collaborated with Gary Roubin, then a cardiologist at Emory, to develop a coronary stent based on Gianturco’s initial wire coil designs. After Gruentzig’s untimely death in a plane crash, Roubin continued the development, and after about a year started testing a balloon-expandable flexible coil stent (Gianturco-Roubin Flex-Stent). This stent was tested to treat acute vessel closure, a medical emergency, following balloon angioplasty, and gained FDA approval in early 1993 for this indication.

**The Wallstent and Multilink stent.** Ulrich Sigwart was a cardiologist working at the Centre Hospitalier Universitaire Vaudois in Lausanne, Switzerland. In the early 1980s, Sigwart built self-expanding stents from an elastic wire braid, inspired by cylindrical Chinese fingertraps made from woven strips of bamboo. Initial prototypes and animal studies were supported by the University’s experimental surgery department and a grant from the Swiss National Fund. In 1985, Sigwart partnered with MedInvent, a small private medical device company in Switzerland, to provide additional supplies and manufacturing and engineering support for the stent, which was later named the Wallstent. This work led to the first stent placement in the coronary arteries of patients in Europe [23]. In 1986, MedInvent was acquired by Schneider, a subsidiary of Pfizer, but the company put development of the Wallstent on hold due to liability concerns stemming from catastrophic mechanical failures in a separate implantable cardiac product, the Bjork-Shiley heart valve.

With Wallstent development at a standstill, Sigwart began to work in 1989 with a small team at ACS, a private catheter focused company, to pursue a balloon-expandable stent, leading to the MultiLink stent. In 1993, the first Multilink stent was implanted in a patient in London. In 1997, it was approved by the FDA and quickly gained market dominance due to its improved steerability.

**Other stents.** These early devices led to a much better understanding of what factors were needed to provide deliverability, flexibility, and radial strength. A second wave of coronary artery stent designs were commercialized more expeditiously due in part to regulatory approval pathways blazed by the earliest innovators. For example, whereas the FDA required the Palmaz-Schatz stent to be tested in the peripheral circulation before being applied to coronary arteries, this hurdle was not imposed on any other designs. Independent US-based inventor Dominic Wiktor developed a stent with Medtronic that was FDA-approved in June 1997. Advanced Vascular Engineering’s stent was approved in December 1997; the company was subsequently acquired by Medtronic in 1998. European interventional radiologist Ernst Strecker developed stents for peripheral use in the 1980s and partnered with Boston Scientific—which went public in 1992—to create a self-expanding coronary artery stent (approved late 1998). More recently, stent innovation has prioritized drug-eluting stents and bio-absorbable stents, which can be traced to work by Richard Stack at Duke University in the early 1980s.

### Role of individual inventors

We found wide agreement that individual inventors played the primary role in early development (Table 1). When describing the origins of this transformative device, respondents commonly pointed to the key contributions of Drs. Palmaz, Schatz, Gianturco, Roubin, and Sigwart.

**Table 1:** Representative Quotations in Key Subject Areas.

Thematic area	Illustrative remarks
Motivation	“My involvement was driven purely and simply by a clinical imperative at the time.” [*Physician-innovator*]
	“It was clear … that we had a huge shortcoming with [Gruentzig’s] method and that was his acute closure that led to tremendous amount of myocardial infarction emergency surgery.” [*Physician-innovator*]
	“I felt very strongly being an operator. I wasn’t just an inventor. I was an operator.” [*Physician-innovator*]
Obstacles to progress	“We had to be very hard headed to accept all that rejection because it was systematic and relentless.” [*Physician-innovator*]
Contributors to early success	“We spent a lot more time on the design and a lot more time proving that it worked. All the others were just kind of wham-bam. ‘Let’s get it out there as fast as we can’ but without any data.” [*Physician-innovator*]
	“We are more aggressive with filing patents today than we were back then, but in those days, we didn’t file any patents. We just kept our nose to the grindstone and kept things moving.” [*Company-based innovator*]
Risk and investment	“When the thing is disruptive and totally outlandish, the companies stay away from it.” [*Physician-innovator*]
	“My first reaction when I was told what it was and what I should be thinking about designing was well that’s a stupid idea. We’ve got diseased arteries that are full of stuff already, why would we want to put in a piece of a metal that’s going to be lifetime liability for us?” [*Company-based innovator*]
Collaboration	“So many, many months and changes and design went by working with the engineers from [company]” [*Physician-innovator*]
	“From an engineering product development perspective, they are extraordinary.” [*Physician-innovator*]
	“When [company] took over we just basically showed the engineers what we wanted and that was it.” [*Physician-innovator*]
	“I mean he had a lot of clinical issues that he saw that needed to be solved and he needed some help in doing that, and we helped him in any way we could. It was just a nice partnership.” [*Company-based innovator*]
Role of intellectual property	“The impetus was to publish and it seems quaint now and maybe stupid, but we didn’t give much thought to patenting.” [*Physician-innovator*]
	“The patents of course are critical because no company wants to invest unless they have some IP.” [*Company-based innovator*]
	“In those days, we were for a couple of years the number one patenting company in the nation, if not the world.” [*Company-based innovator*]

These key innovators (Gianturco was deceased) were early adopters of coronary artery angioplasty and had first-hand exposure to clinical problems related to the technique, most notably post-angioplasty restenosis. Some conceived of the stent as an alternative to angioplasty, while others viewed it as a way of preventing abrupt artery closure that occurred after vessel dissection from treatment with a balloon catheter, which was a medical emergency. As one inventor noted, “I found that balloon angioplasty was unpredictable, and I said we must find some sort of endoluminal support.” Later, use of the stent in preventing restenosis and increasing the durability of percutaneous revascularization procedures was also realized.

The inventors did substantial work in developing the technology. After failing to secure industry partners in the early 1980s, Palmaz progressed through animal studies himself and filed the Investigational Device Exemption to begin human testing. Similarly, Sigwart engaged in prototype development and animal testing of the Wallstent on his own time.

All of these innovators faced substantial skepticism, particularly from the medical device industry and grant funding agencies. As one inventor said, “People hated the stent; they hated it. It was incredible. When you would go to a company and would show them the stent in the early ‘80’s... immediately you can see their face, they start rolling their eyes, and kind of making scowls. There was something about stents that everybody disliked.” The Veterans Administration’s rejections of Palmaz’s grant applications to fund his work compelled him to find private funding through Romano and Schatz. Despite early reports of stent thrombosis as high as 24% [24], innovators such as Palmaz and Schatz remained resolute in the belief that the stent technology when deployed correctly and with the recommended course of antithrombotic medication was safe and effective. They were buoyed by support among their colleagues in the interventional radiology and cardiology communities, who saw the need and importance of such a technology, and who provided support and collaboration.

### Role of industry

Most interviewees described the entry of large medical device companies after stent prototypes had been sufficiently developed and tested in laboratory and animal trials. Risk was cited as a primary factor that hindered earlier industry involvement. According to interviewees, companies believed it was physiologically incompatible to implant prosthetic material in the coronary circulation. Companies also had legal concerns. The Bjork-Shiley mechanical heart valve had been recalled around that time for safety reasons prompting Pfizer to halt development of Sigwart’s first stent product. Cook Inc. was concerned about legal risks related to the potential failure of implantable cardiac devices, necessitating Roubin to personally seek the initial Investigational Device Exemption application to begin testing his stent in humans. Given the invasiveness of the technology, there was also significant concern regarding the FDA approval process and the “difficult regulatory environment.” Finally, according to interviewees, many companies perceived substantial business risks. According to one inventor, consultants from McKinsey & Co. provided a strong recommendation to Johnson & Johnson against investing in the Palmaz-Schatz stent believing the market size to be too small.

When they became involved, medical device companies provided financial resources and engineering to test design hypotheses of physician-innovators. The company’s engineers also supported manufacturability. However, individual inventors reported that they accounted for ease of manufacturing in their initial prototype designs. The Palmaz-Schatz laboratory-produced prototype was essentially manufacture-ready when Johnson & Johnson became involved.

Second, medical device companies provided necessary support in organizing clinical trials and negotiating the FDA approval process. Organizing the randomized trials and FDA premarket authorizations took 7–8 years to complete. The inventors estimated that the companies invested in the range of $100–500 million in the processes leading to device approval, earning revenues surpassing that investment within a year or two after the devices were approved. Lastly, device companies provided existing sales channels to deploy the technology, although innovators helped convince other clinicians to adopt the technology.

### Role of intellectual property

We found that ownership of intellectual property played little role in incentivizing initial innovation in this field. No key inventors initially sought out patents after developing their stents citing a lack of expertise, funding limitations and a philosophical commitment to research dissemination. Our interviewees each said that the potential profitability of the resulting products was not an important consideration.

The key innovators in our study considered themselves uninformed about patents. One inventor said, “I was completely naive in the area and the only thing I wanted was to get the scaffold into the angioplasty.” Another inventor first broached the subject of patenting with his university, but reports that he was told by university officers that his work was not patentable. Without patent protection, an inventor presented his work to the public at a national conference, later leading to a loss of European rights. A third inventor did not initially apply for a patent on his work; rather, intellectual property protection was sought first by the company he was associated with only after he signed a contract releasing the technology rights to them.

Patents ultimately became paramount in the context of the larger corporations that later became involved in stent commercialization. After Palmaz, Schatz, and Romano formed EGP, the partnership then applied and paid for its own patent [25]. Johnson & Johnson later expended substantial resources, in the words of one interviewee, “expanding the patent limits.” The patent record became crucial as these larger companies engaged in litigation with one another. By 2002, multiple lawsuits between Johnson & Johnson, Cordis, Medtronic and Boston Scientific about which had priority to overlapping designs of their different versions of coronary artery stents resulted in billions of dollars in damages and fees [26]. In one judgment, Medtronic and Boston Scientific paid Johnson & Johnson $1.2 billion for patent infringement on the Palmaz-Schatz stent.

A lax posture towards patents excluded some inventors and key contributors from financial rewards. For example, the University of Texas, which financially supported and provided laboratory space for important early proof-of-concept experiments conducted by Palmaz, declined to invest the resources to patent the discovery. Ultimately, Palmaz offered them a 3% share of his royalties, which has since led to about $10–$30 million in total payments to the university. Roubin and Emory University similarly received no royalties from his work or the testing that occurred in university laboratories, although Cook later provided Roubin with a substantial financial gift to recognize his contribution. Without patents, Sigwart and his institution received no royalties from his original innovations, even after the technology was sold to Pfizer. This experience led him to change his approach towards intellectual property in his subsequent collaboration with ACS.

### Other key characteristics of the inventors and inventive process

Key inventors were all physicians directly exposed to the clinical problems they were trying to address. Most interviewees specifically remarked on these inventors’ aptitude and vision, such as their ability to recognize potential innovative solutions to emerging clinical problems such as coronary artery restenosis. Interviewees also pointed to the inventors’ resiliency despite considerable resistance from the medical community. Another common quality was that inventors were seen as risk-takers, in contrast to the widespread perceptions that the companies were risk-averse. Interestingly, a background in engineering was not necessary. All individual inventors were able to directly develop prototypes from raw materials and deploy them on animals.

Collaborative work was central to the early development process. For the Palmaz-Schatz and Gianturco-Roubin stents, each inventor brought different contributions to building the device. Interviewees cited the importance of collaborations between the academic inventors and their colleagues in medical device companies for engineering support. Other unofficial collaborations also helped move the development process forward. For example, Palmaz received early assistance from an engineer not affiliated with his academic medical center in learning about certain manufacturing processes such as laser etching that he could use to produce a prototype of his stent concept [27]. Interviewees also recalled the inventors discussing their work at national professional meetings, and the importance of these open brainstorming sessions in facilitating progress of the individual projects.

## Discussion

Consistent with other single-perspective historical analyses of coronary artery stents [28], [29], our qualitative study found that much of the work in developing coronary stents was pioneered by individual inventors, not only in generating ideas in the face of substantial skepticism, but also in prototype development and early testing. These individual inventors were motivated by the desire to address a pressing clinical problem to which they had direct exposure.

Their work was facilitated by supportive atmospheres within academic or other research environments that allowed freedom for collaborations and provided resources for proof-of-concept testing. Larger companies became involved at a later stage, contributing manufacturing support, resources for broad clinical testing, and regulatory approval.

Our findings suggest a conceptual model in which transformative medical device innovation arose from three primary factors: (1) unmet clinical need, (2) independent innovators with the insight, motivation, and ability to address that need, and (3) support that allowed the innovators to push their solutions past skepticism and through proof-of-concept testing. In the case of coronary artery stents, the clinical imperative arose from complications of the emergence of balloon angioplasty, as well as from the desire to apply existing experimentation with peripheral artery stents to relieve coronary artery blockages. The fact that many of the key innovators were physicians who could draw on their direct experience with patients turned out to be central to recognizing and addressing this clinical need. In the early stages of development, innovators received their primary support from academic research centers and mentors, from small companies with close ties to innovative researchers, and in the case of the Palmaz-Schatz stent, from a forward-thinking angel investor with no pre-existing ties to the medical industry [30].

It is widely argued that there is a positive association between profit expectation and innovative activity [31]. However, most of the key inventors of coronary artery stents reported to us that they did not have any profit expectations, and in fact did not initially seek out patents. Indeed, many of these innovators openly shared their work with colleagues, as well as in publications and at national meetings. In some cases, this led to design improvements. In others, there was a direct loss of patent rights in certain jurisdictions.

In the case of device development, industry representatives have often referred to themselves as the primary sources of new products [22]. Others point to the primacy of small companies in the device innovation process [32]. However, the evidence we have collected indicates that individual innovators supported by academic medical centers were the primary source of development for this transformative device. Academic physicians did not just generate the ideas, or conduct basic research that would lay the groundwork for subsequent discoveries. They completed prototype development and conducted clinical testing up to the point of initial FDA approval of human trials.

Given the perception of the innovators we spoke with that established companies and venture capitalists were—and remain—generally risk-averse regarding highest risk and most innovative medical technology, funding for such work outside of these channels is critical. Thus, supporting basic research in new devices through the NIH and facilitating the efforts of innovators who seek to move their discoveries out of the academic setting are likely to have the greatest impact in generating breakthrough discoveries. Government funding for science has slowed in recent years, and faces substantial budget cuts in the future as well [33]. By contrast, policies that have been proposed or enacted in recent years—adjusting FDA regulatory practices or providing additional resources to large manufacturers, such as by repealing the excise tax on approved medical devices in the 2010 Patient Protection and Affordable Care Act—are unlikely to have a substantial impact on breakthrough discoveries, despite the claims made by proponents of these strategies [34].

In addition, the primacy of physician-innovators in our study reinforces the need to foster relationships between academic researchers and device industry that can facilitate uptake of transformative ideas and prototypes [35], [36]. As one physician-innovator noted, “The first thing we do when we get a great idea now is get it outside [the academic setting] and get it commercialized.” Physician-innovators with existing relationships with companies were able to more speedily advance their work (Gianturco/Roubin and Cook Inc.) as compared to those who did not (Palmaz). To attract industry interest, Palmaz had to demonstrate more clinical efficacy in animal studies and human case studies than did the innovators allied with Cook Inc., who already had a longstanding relationship.

Finally, we found that patents played a limited role in the innovation of the coronary artery stent. Notably, in recent years, physician-innovators and academic medical centers have shown greater propensity towards obtaining patents [37]. Some reports have suggested that the proliferation of patents might be hindering transformative innovation [38], and provided specific examples where this has been the case in the medical device market [39]. Innovators in our sample pointed to current-day patenting trends as harmful to the essential collaborative relationships they developed during their work on coronary artery stents, and blamed these trends on certain university technology transfer offices seeking greater control over patent rights or insisting on burdensome licensing agreements [40]. There remains significant uncertainty regarding the ownership of patentable improvements on original designs licensed from different parties. Many licensing agreements clearly specify ownership relating to improvements. However, inattention to patents in the stent case also hindered the protection of the technology. Further research is required to determine whether increased attention to patents and revenue generation on the part of physician-innovators and academic research centers today does indeed contribute to reduced innovation. If so, alternative mechanisms must be identified to facilitate the continued development of transformative medical technologies.
